# Factors contributing to the recalcitrance of herbaceous dicotyledons (forbs) to enzymatic deconstruction

**DOI:** 10.1186/1754-6834-7-52

**Published:** 2014-04-05

**Authors:** Dina Jabbour, Evan R Angelos, Achira Mukhopadhyay, Alec Womboldt, Melissa S Borrusch, Jonathan D Walton

**Affiliations:** 1 Department of Energy, Great Lakes Bioenergy Research Center, 1129 Farm Lane, Michigan State University, East Lansing, MI 48824, USA; 2 Department of Energy, Plant Research Laboratory, 612 Wilson Road, Michigan State University, East Lansing, MI 48824, USA

**Keywords:** Milkweed, Goldenrod, Queen Anne’s lace, Lamb’s quarters, Cellulase, Biofuels, Pretreatment, Alkaline hydrogen peroxide, AFEX

## Abstract

**Background:**

Many different feedstocks are under consideration for the practical production of biofuels from lignocellulosic materials. The best choice under any particular combination of economic, agronomic, and environmental conditions depends on multiple factors. The use of old fields, restored prairie, or marginal lands to grow biofuel feedstocks offers several potential benefits including minimal agronomic inputs, reduced competition with food production, and high biodiversity. However, a major component of such landscapes is often herbaceous dicotyledonous plants, also known as forbs. The potential and obstacles of using forbs as biofuel feedstocks compared to the more frequently considered grasses and woody plants are poorly understood.

**Results:**

The factors that contribute to the yield of fermentable sugars from four representative forbs were studied in comparison with corn stover. The forbs chosen for the study were lamb’s quarters (*Chenopodium album*), goldenrod (*Solidago canadensis*), milkweed (*Asclepias syriaca*), and Queen Anne’s lace (*Daucus carota*). These plants are taxonomically diverse, widely distributed in northern temperate regions including the continental United States, and are weedy but not invasive. All of the forbs had lower total glucose (Glc) content from all sources (cell walls, sucrose, starch, glucosides, and free Glc) compared to corn stover (range 16.2 to 23.0% on a dry weight basis compared to 39.2% for corn stover). When digested with commercial enzyme mixtures after alkaline pretreatment, yields of Glc as a percentage of total Glc were lower for the forbs compared to corn stover. Enzyme inhibition by water-extractable compounds was not a significant contributor to the lower yields. Based on experiments with optimized cocktails of pure glycosyl hydrolases, enzyme imbalance probably accounted for much of the lower yields. Addition of xyloglucanase and α-xylosidase, two enzymes targeting Glc-containing polysaccharides that are more abundant in dicotyledonous plants compared to grasses, enhanced Glc yields from lamb’s quarters, but Glc yields were still lower than from corn stover.

**Conclusion:**

The potential utilization of forb-rich plant communities as biofuel feedstocks must take into account their lower Glc content compared to grasses such as corn stover. Furthermore, new enzyme mixtures tailored to the different cell wall composition of forbs will have to be developed.

## Background

Transportation fuels from lignocellulosic biomass have the potential to contribute to national and regional energy independence, improved economics, and environmental sustainability [1-3]. From the point of view of sustainability, potential biomass feedstocks differ considerably from each other in their requirements for chemical and energy inputs and their positive and negative contributions to environmental health [4]. Conventional monoculture crops are high input and low diversity, whereas feedstocks composed of mixed native or naturalized plants growing with minimal human intervention in either undisturbed or former agricultural land are low input and high diversity. Low-input high-diversity agronomic landscapes include native and restored prairie, marginal lands, and old fields.

A number of studies have addressed the economic and environmental implications of producing biomass for bioenergy from low-input high-diversity landscapes [5-14]. Garlock *et al.*[7] found that fermentable sugar yields positively correlated with the percent composition of grasses compared to forbs in early successional old field communities comprising 7 to 14 species. This was attributed to both the higher glucan content of grass cell walls and the greater enzymatic conversion efficiency of grass biomass. However, the inherent species complexity of natural plant communities makes it challenging to control for differences between species within the two groups (grasses and forbs) and to elucidate the underlying reasons for the apparent superiority of grasses over forbs. In order to minimize some of the variability innate in mixed communities, we have compared four individual forb species for yields of fermentable sugars, using corn stover (CS) as a benchmark. Factors studied included total glucose (Glc) content, response to pretreatments, presence of enzyme inhibitors, and enzymatic digestibility.

## Results

The four species of forbs used in this study were milkweed (MW) (*Asclepias syriaca*), Queen Anne’s lace (QA) (*Daucus carota*), lamb’s quarters (LQ) (*Chenopodium album*), and goldenrod (GR) (*Solidago canadensis*). These species were chosen because they are taxonomically diverse, widely distributed in northern temperate regions including the continental United States, frequent components of old fields and marginal lands, and weedy but not invasive

### Composition analysis

The plant materials were ground but not otherwise treated or washed before compositional analysis (Table 1). Therefore, the analysis includes not just structural (cell wall) sugars but also free sugars, sugar nucleotides, sucrose, starch, and glycosides. This is a more realistic estimate of the actual material that would be encountered in a lignocellulosic ethanol facility than if only the sugars present in structural macromolecules were analyzed. Neutral sugars (Glc, Xyl [xylose], Gal [galactose], Ara [arabinose] + Man [mannose]) comprised 66.8% of the total dry weight of CS whereas the forbs were very similar to each other, ranging from 35% to 36.4%. Structural Glc made the greatest contribution to total Glc across all plants, although the forbs did differ from each other in their levels of sucrose, starch, and free Glc. In regard to the content from all sources of Glc, the most valuable fermentable sugar, CS was much higher than any of the forbs (39.2% versus 16.2 to 23.0%).

**Table 1:** Composition of plant materials

	**Protein**	**Glc**	**Xyl**	**Gal**	**Ara + Man**^**b**^	**Sucrose**	**Starch**	**Free Glc**	**Total neutral sugars**
Goldenrod (GR)	10.3 ± 1.1	214.5 ± 2.7	47.4 ± 0.7	11.7 ± 2.2	18.3 ± 0.3	38.3 ± 2.5	6.0 ± 1.0	13.8 ± 1.7	350
Lamb’s quarters (LQ)	18.7 ± 7.3	161.6 ± 1.7	22.3 ± 0.3	13.7 ± 1.1	23.4 ± 0.6	46.0 ± 3.6	25.6 ± 1.1	33.4 ± 3.5	356
Milkweed (MW)	12.6 ± 7.9	201.8 ± 7.6	26.8 ± 1.2	21.2 ± 1.2	14.9 ± 0.5	67.8 ± 3.4	10.9 ± 1.2	24.6 ± 1.3	368
Queen Anne’s lace (QA)	12.7 ± 1.2	230.0 ± 18.6	48.7 ± 2.0	13.2 ± 2.0	18.7 ± 0.5	31.2 ± 4.0	4.5 ± 1.3	17.6 ± 1.5	364
Corn stover^a^ (CS)	4.0 ± 0.7	391.5 ± 0.4	194.7 ± 10.9	9.4 ± 2.3	33.3 ± 5.3	11.5 ± 0.4	11.8 ± 0.8	16.0 ± 1.8	668

^a^CS data are from [19,23].

^b^The HPLC method did not resolve Ara and Man.

Materials were dried but unwashed before analysis, and therefore the values include contributions from non-cell wall components such as starch, glycosides, sucrose, and free Glc. All values are mg/g dry weight ± 1 SD (n = 3).

Ara, arabinose; Gal, galactose; Glc, glucose; Man, mannose; Xyl, xylose.

### Pretreatments

**Figure 1:**
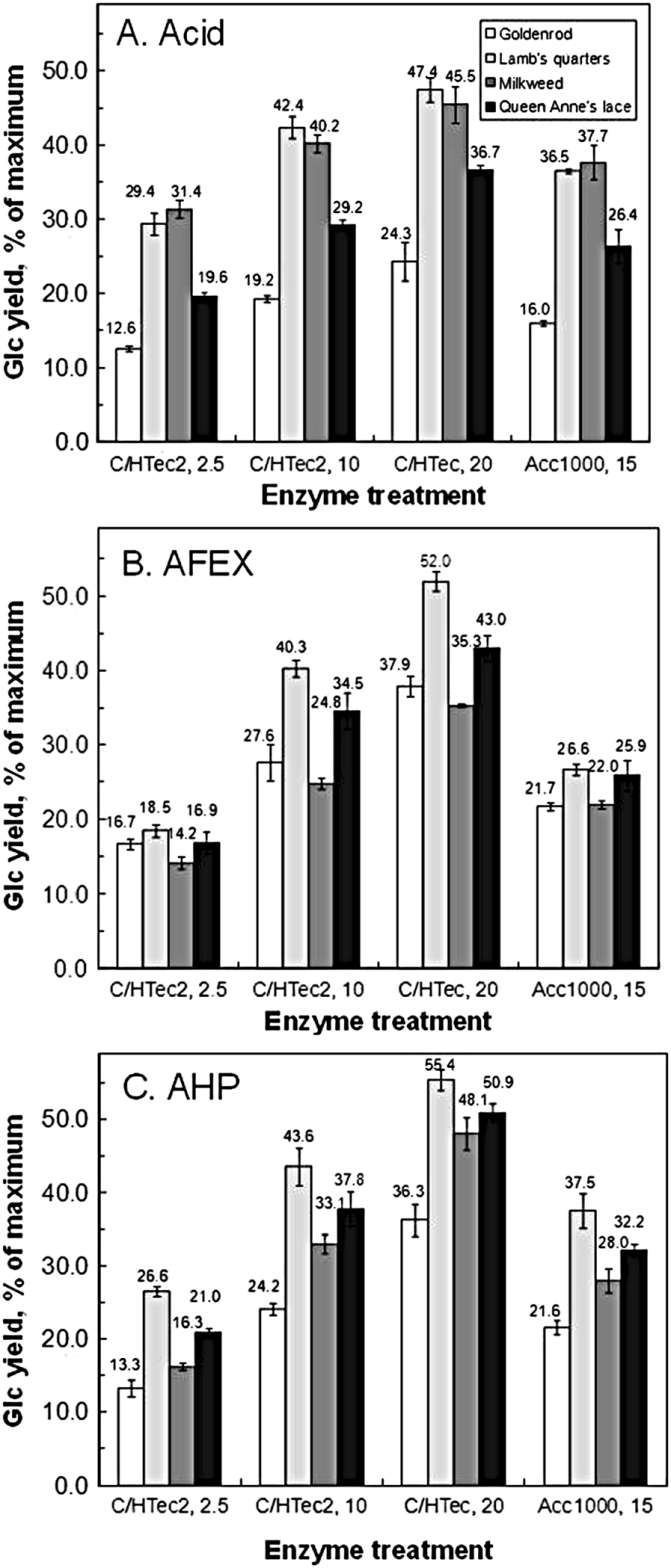
**Comparison of three pretreatments on subsequent enzyme hydrolysis of four species of forbs. A**. Dilute acid. **B**. AFEX. **C**. AHP. C/HTec2 refers to a 3:1 mixture of Cellic™ CTec2 and HTec2. Acc1000 is Accellerase 1000. Numbers after the enzyme names indicate the loadings in mg protein/gm glucan. Biomass loading was 2 mg glucan/ml, incubation temperature was 50°C, and incubation time was 48 hours. AFEX, ammonia fiber expansion; AHP, alkaline hydrogen peroxide; Glc, total glucose.

The four forbs were pretreated with dilute acid, ammonia fiber expansion (AFEX), or alkaline hydrogen peroxide (AHP). Four concentrations of acid (H_2_SO_4_) and twelve AFEX treatments were compared on goldenrod (GR) alone. Materials were subsequently digested with a 3:1 ratio of Cellic™ CTec2 plus HTec2 (abbreviated C/HTec2) at a loading of 30 mg/g glucan for 96 hours. The acid-treated material was neutralized with NaOH but not washed after pretreatment. Among acid treatments, the highest concentration tested (1.5%) gave the best yield, and among AFEX conditions, an ammonia loading of 1.5:1 at 100% moisture (140°C) for 15 minutes gave the best yields (data not shown). All four forbs were then subjected to these same acid and AFEX conditions, plus alkaline hydrogen peroxide (AHP), and then digested with three loadings of C/HTec2 and one loading of Accellerase™ 1000Averaged across all enzyme loadings and plants, AHP performed better than acid, which performed better than AFEX. Compared across enzyme loadings, AFEX performed the best on GR followed closely by AHP, acid performed the best on MW, and AHP performed the best on LQ and QA (Figure 1). Due to its overall good performance and ease of execution (it is performed at room temperature and atmospheric pressure in inexpensive containers) [15,16], AHP-pretreated material was used in subsequent experiments.

### Enzymatic hydrolysis

**Figure 2:**
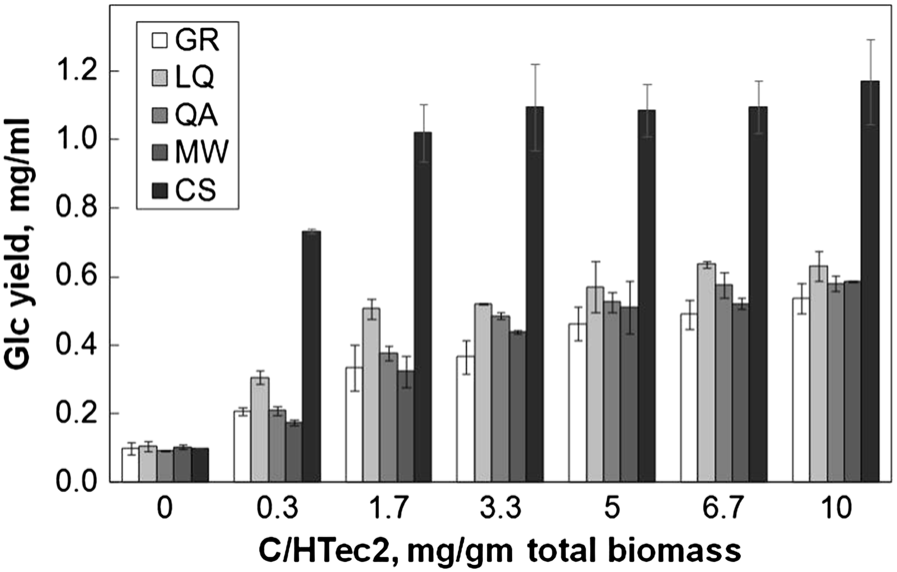
**Glc yields at constant biomass loading from pretreated forbs and corn stover.** The pretreatment was AHP, biomass loading was 3 mg/ml, incubation time was 48 hours, and incubation temperature was 50°C. AHP, alkaline hydrogen peroxide; CS, corn stover; Glc, total glucose; GR, goldenrod; LQ, lamb’s quarters; MW, milkweed; QA, Queen Anne’s lace.

In the initial enzymatic hydrolysis experiment, total biomass loading was kept constant at 3 mg/ml. The Glc yield from CS was 1.17 mg Glc/ml (Figure 2), which is about 91% of the maximal possible. The Glc yields from the forbs species were considerably lower, with the maximum yields from GR, LQ, QA, and MW being 0.54 mg/ml, 0.64 mg/ml, 0.58 mg/ml, and 0.59 mg/ml, respectively (Figure 2).

**Figure 3:**
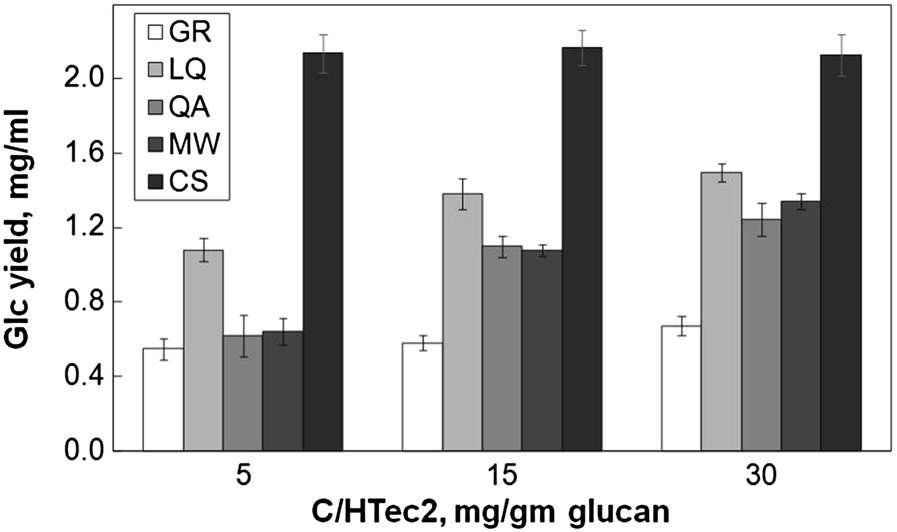
**Glc yields at constant glucan loading from forbs and CS.** The values are reported as mg/ml in order to facilitate comparison with the experiment shown in Figure 2. Glucan loading was 2 mg/ml, which corresponds to 100% of maximum theoretical yield in other experiments. The pretreatment was AHP, incubation time was 48 hours, and incubation temperature was 50°C. AHP, alkaline hydrogen peroxide; CS, corn stover; Glc, total glucose; GR, goldenrod; LQ, lamb’s quarters; MW, milkweed; QA, Queen Anne’s lace.

Enzymatic hydrolysis was then compared on an equal glucan loading (Figure 3). After 48 hours of hydrolysis, apparent Glc yields from CS were more than 100%. This apparent yield of >100% was probably due to a combination of factors, including experimental error in the measurement of Glc content and of Glc yields, and Glc contributed by the enzyme cocktail. Wolfrum *et al.*[17] reported a similar >100% yield and discussed other possible explanations. Of the forb species, LQ was the most digestible, achieving 74% conversion with the highest C/HTec2 loading (30 mg/gm glucan). At a C/HTec2 loading of 15 mg/gmglucan, Glc yields from LQ, GR, QA, and MW were 65%, 29%, 55%, and 54% of maximal, respectively. That is, even adjusting for differences in Glc content, yields of Glc from the forbs were low compared to CS.

### Preparation and analysis of extractives

Pretreatment of lignocellulosic material results in the formation and/or release of a number of substances inhibitory to enzymes and fermentative microorganisms [18]. As one possible explanation for the lower yields of Glc from the forbs compared to the CS, even when adjusted for their lower Glc content, we examined the possibility that the forbs contain soluble inhibitors of enzymes. Low-molecular weight materials, known as extractives, were prepared by washing the plant materials sequentially with water, ethanol, and acetone. The extractives were tested at equal relative concentrations for their effects on the digestibility of CS. Extractives prepared in the same way from CS were used as a control. The extractives contained significant amounts of Glc in both free and polymeric form, which were subtracted from total Glc yields to calculate the yields from enzymatic digestion of the polymeric, insoluble glucans alone.

**Figure 4:**
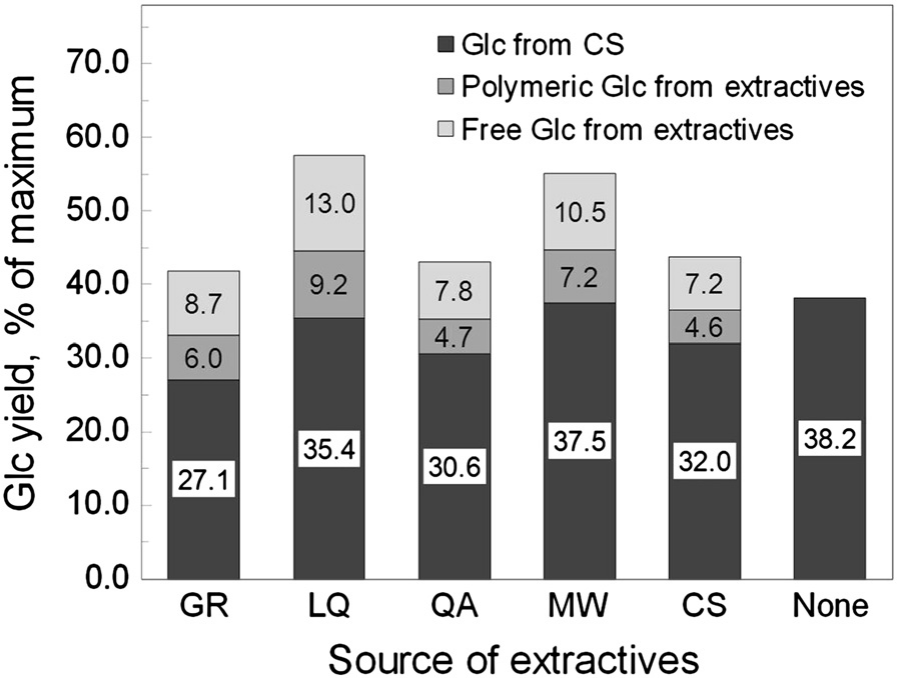
**Effect of extractives from forbs on enzymatic deconstruction of CS.** Water and solvent-soluble materials were extracted from the indicated forbs or from CS. The total heights of the bars indicate the total Glc measured in each sample following enzymatic digestion of each CS sample by C/HTec2, added at 10 mg/g glucan for 48 hours at 50°C. The different shadings indicate the amount of Glc originating from free Glc that was present in the extractives added to each reaction (light bars), the Glc released by the enzymes due to the enzymatic digestion of the CS biomass (black bars), and the Glc released upon digestion of soluble biomass components that were present in the extractives added to the reactions (medium grey bars). Reduction of polymeric Glc yields from the CS (black bars) compared to the no-extractives control (38.2% Glc yield) indicates the degree of enzyme inhibition by the extractives from each plant species. The differences in digestibility of GR, QA, and CS were significantly different from the CS alone at *P* < 0.05. AHP, alkaline hydrogen peroxide; CS, corn stover; Glc, total glucose; GR, goldenrod; LQ, lamb’s quarters; MW, milkweed; QA, Queen Anne’s lace.

Comparison of the yields of Glc from CS in the presence of inhibitors, compared to CS alone or CS + extractives from corn, indicated that forbs extractives caused some inhibition (indicated by the relative heights of the black bars in Figure 4). Soluble extracts of CS inhibited yields of Glc from CS by 16.2%. GR extracts were the most inhibitory (29.1% inhibition). The other forbs were intermediate, LQ and MW being less inhibitory than CS and QA slightly more inhibitory (Figure 4).

### Optimization of synthetic 8-component enzyme mixtures

Even taking into account the lower Glc content of the forbs and the slight inhibition by their soluble components, yields of Glc from the forbs were still lower than from CS. A possible explanation for this is that the enzymes found in C/HTec2 are inappropriate in content or in proportions for the digestion of the cell walls of herbaceous dicotyledonous plants. To test this, enzyme optimization experiments were performed. Synthetic mixtures of eight ’core’ enzymes (BG, EG1, CBH1, CBH2, GH61, BX, EX2 and EX3) were optimized [19,20]. A minimum proportion of 5% was set as a lower limit for all enzymes.

In the case of CS, the following proportions of an 8-component synthetic enzyme mixture resulted in the highest Glc yield of approximately 75% after 48 hours: 30% CBH1, 20% EG1, 20% GH61, 5% BG, 5% CBH2, 5% BX, 5% EX2, and 10% EX3. The resulting model was statistically significant. In the case of the forbs, under the same experimental design conditions, Glc yields never exceeded approximately 20 to 30% at any enzyme combination, and as a result no statistically significant model of optimized proportions could be determined (data not shown). Apparently, although C/HTec2 is suitable for reasonable yields from forbs as well as CS, mixtures containing only these eight enzymes in any proportions are insufficient for the hydrolysis of forbs. Other enzymes, which are present at least in part in C/HTec2, are necessary for hydrolysis of the cell walls of herbaceous dicotyledons.

### Accessory enzymes

C/HTec2 could release more than 75% of the Glc from some forbs (Figure 3), but the optimized 8-component could not release more than 30%. One possible explanation for this result is that forbs, but not CS, require additional accessory enzymes that are present in C/HTec2 but not in the 8-component mixture. In an attempt to identify what these hypothetical enzymes might be, additional accessory enzymes were tested by supplementation of the 8-component mixture. As dicotyledonous plants contain more pectin than cereal cell walls, and modification of pectin composition has been shown to enhance Glc release [21], supplementation with Multifect™ Pectinase was first tested. Multifect™ Pectinase, derived from *Aspergillus niger*, contains more than 130 proteins including numerous pectinases of diverse specificities [22]. However, in no experiment did Multifect™ Pectinase enhance either Glc or Xyl yields from any of the forbs, either in combination with the 8-component mixture or in combination with C/HTec2 (data not shown).

**Figure 5:**
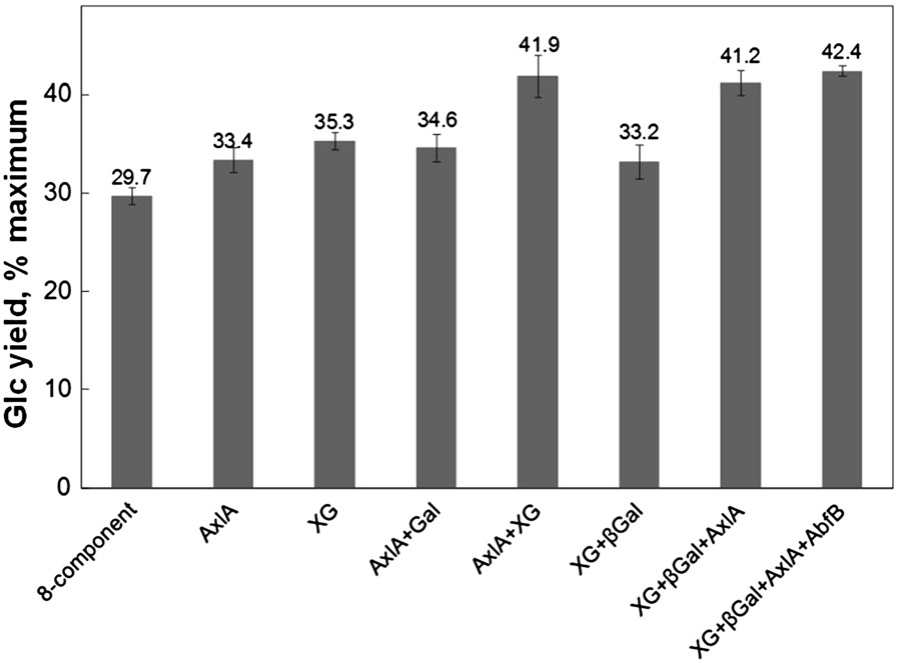
**Effect of accessory enzymes on hydrolysis of lamb’s quarter in combination with the 8-component synthetic mixture**. Enzymes were xyloglucanase (XG), α-xylosidase (AxlA), β-galactosidase (βGal), and α-arabinosidase (AbfB). The 8-component mixture loading was 15 mg/gm glucan and total accessory enzyme loading was 5 mg/gm glucan. Digestion was for 48 hours.

Supplementation of the 8-component mixture with individual enzymes was also attempted. All of the pure enzymes were from *Trichoderma reesei* and expressed in *Pichia pastoris*, except AxlA, which was expressed in *P. pastoris* from an *A. niger* gene [22,23]. LQ was used as the substrate at a loading of 2 mg glucan/ml. Supplementation of an 8-component mixture (in the proportions shown to be optimal for CS) with 5 mg/gm glucan of Abf1, Abf2, AbfB, Cip 1, Cip2, GH12, or β-galactosidase did not enhance Glc yields from LQ, but supplementation with xyloglucanase (XG,Cel74A) and α-xylosidase (AxlA) did enhance yields (Table 1 and Figure 5). AxlA had earlier been shown to enhance Glc yields from LQ in combination with C/HTec2 [23]. Supplementation of the 8-component mixture with both AxlA and XG increased yields of Glc by 12.2%, and further addition of β-galactosidase or AbfB had no effect (Figure 5). These results indicate that one of the enzymes present in C/HTec2 that is important for Glc release from forbs is XG (C/HTec2 lacks AxlA or α-xylosidase activity), but that there are probably others.

## Discussion

There have been relatively few studies exploring the potential utility of herbaceous dicotyledons (forbs) for the production of lignocellulosic biofuels. Past studies have either looked at dicotyledonous crop residues (such as straw from sunflower, soybean, alfalfa, canola, and cotton), or forbs in the context of mixed communities predominantly containing grasses [7,24-31]. To survey the potential of forbs that are likely to be found in northern temperate mixed plant communities such as old fields and marginal lands, and to reduce the complexity of working with a mixture of species, we explored the factors affecting Glc yields from four single species of forbs. CS, a widely used lignocellulosic feedstock, served as a benchmark. The factors considered were sensitivity to pretreatments, content of neutral sugars, presence of enzyme inhibitors, and enzymatic digestibility. We found that yields of Glc from all forbs were consistently much lower than from CS, and that two factors dominated the recalcitrance of forbs: lower Glc content and enzyme imbalance. Our study did not address the possible importance of either lignin content or lignin structure, both of which are known to differ between dicotyledons and grasses [32,33]. The four forbs showed similar trends in regard to glucan content and digestibility even though they were taxonomically diverse, and therefore it should be possible to extrapolate from the results in this paper to other herbaceous dicotyledons.

In regard to the lower Glc content in our experiments, this was probably in part due to the harvest time of the forbs. CS as a lignocellulosic biomass is still a secondary crop to the more valuable grain, and is therefore harvested after the translocatable nutrients in the leaves have been mobilized into the grain. On the other hand, the forbs used in the current work were harvested midseason when they were still actively growing, and therefore were richer in non-structural constituents such as chlorophyll, primary metabolites, and protein. This is evidenced by the protein and soluble sugar content of the forbs compared to CS (Table 1). If the forbs had been harvested in the late autumn, they may have had lower protein and other cytoplasmic contents, and a higher structural polysaccharide content [26].

Determining when might be the best time to harvest forbs for bioethanol production remains to be established. Younger plants tend to have a lower lignin content, which is positively correlated with enhanced enzyme digestibility, but younger plants are also smaller and their higher mineral nutrient content compared to senescent plants would result in greater loss of soil nutrients from the harvested fields. Various agronomic practices have been envisioned for optimal harvesting of forb-rich landscapes such as marginal lands, restored prairie, and old fields. These include harvesting once every few years, once in midseason, once in late autumn, or multiple times throughout the season, and the best choice is dependent on multiple factors [13,24]. Furthermore, agronomic optimization would probably be different for different forbs. For example, because GR and MW are perennials and therefore regrow from the roots each year, a harvest date after the nutrients have been mobilized into the roots would be preferable. On the other hand, if an annual such as LQ were harvested midseason before it sets seed, it could not self-propagate the following season. Yet a different harvest strategy might be preferred for biennials such as QA. In a real-world situation, for example, old fields containing a variety of species, harvest time would have to be a compromise between these factors.

In regard to susceptibility to enzyme digestion, our results indicated that current commercial enzymes are maladapted to forbs. This is not surprising in light of the known differences in cell wall structure between forbs and grasses, and the fact that recent efforts on commercial enzyme improvement have focused on acid-treated CS as the preferred substrate [34].

We had previously developed a mixture of eight purified cell-wall degrading enzymes that could reasonably match commercial enzyme mixtures [19,20]. However, no combination of the same core enzymes was effective on forbs. In an attempt to rectify the poor behavior of the 8-component mixture, additional enzymes were tested. Ax1A, an enzyme lacking in *T. reesei*[22], and XG (Cel74A) together increased Glc yields from GR from 29.7% to 41.9%, which is consistent with the known higher levels of xyloglucan in dicotyledons compared to grasses. This result suggests that there is promise for developing enzyme mixtures adapted to forbs, but also indicates that additional, unknown enzymes will be required to match the performance of commercial mixtures, such as C/HTec2, on CS.

## Conclusions

As naturally occurring, low-input plants, forbs offer distinct advantages as a source of biomass for conversion to biofuels. However, their effective use will require accomodation for their particular properties, which include lower Glc content and poor response to existing enzyme cocktails.

## Methods

### Plant materials, harvest, and preparation

Samples of CS (*Zea mays* Pioneer hybrid 36H56), harvested in the autumn after drydown, was provided by the Great Lakes Bioenergy research center (GLBRC). Samples of MW, QA, and LQ were collected locally from roadsides and abandoned farmland in mid-August, 2010. A sample of GR was obtained from an old field near Kellogg Biological Field Station, Hickory Corners, MI, United States. All above-ground parts of the plants were used.

The plant materials were dried at 50°C for 48 hours and ground with a Christy & Norris 8-inch Lab Mill with a 1-mm screen (Christy-Turner Ltd., Suffolk, United Kingdom). The material was further ground in a Wiley mill (Thomas Scientific, Swedesboro, NJ, United States) to pass through a 0.5 mm screen. All of the starting material was processed through the mill; no material was discarded. Ground samples were stored in sealed containers at room temperature.

### Pretreatments

For dilute acid pretreatment, H_2_SO_4_ concentrations were 0, 0.2, 0.6, and 1.5% (v/v). Samples were autoclaved in the acid at 121°C for 30 minutes, cooled, neutralized with NaOH, and lyophilized. Twelve AFEX conditions were compared, with ammonia loadings of 1:1, 1.5:1, or 2:1; moisture contents of 60%, 100%, or 150%; temperatures of 90°C or 140°C; and times of 15 or 30 minutes [35].

For AHP pretreatment, a solution of H_2_O_2_ diluted from a 30% stock solution (J.T. Baker, ACS Reagent Grade, Fisher Scientific, Pittsburgh, PA, United States) was titrated to pH 11.5 with 5 M NaOH and then mixed with the biomass in a ratio of 0.25 g H_2_O_2_/g biomass. Samples were pretreated for 72 hours at room temperature (21°C) with rotary shaking at 90 rpm. After pretreatment, the pH of the suspension was adjusted to 7.0 with concentrated HCl, treated with catalase to break down residual H_2_O_2_, heated to 90°C for 15 minutes to inactivate the catalase, and lyophilized [15,16]. Because the samples were not washed after pretreatment, the glucan content used for calculating enzyme loadings were adjusted for the weight of the salts resulting from the dilute acid and AHP pretreatments and neutralizations.

### Compositional analysis

Dried, ground, unwashed biomass was subjected to a two-step hydrolysis with sulfuric acid and the sugars quantitated by HPLC using an SP0180 column (Showa-Denko America, Inc., NY, United States) at 85°C with water as mobile phase [36]. This method resolved Glc, Xyl, and Gal, but not Ara and Man from each other. Because the biomass was not washed, the total Glc content included contributions not just from structural polysaccharides but also free Glc, sucrose, glucosides, and starch. Free Glc, sucrose, and starch before acid hydrolysis were assayed as described [37].

The protein content of the plant materials was measured by suspending 100 mg of unwashed biomass in 1.5 ml water and mixing for 1 hour. After centrifugation, the supernatants were assayed for total protein by the method of Bradford [38] with bovine immunoglobulin as the standard.

### Enzymatic hydrolysis

Biomass substrates were suspended at a concentration of 2 mg glucan/ml in 50 mM sodium citrate buffer, pH 5.0, plus cycloheximide and tetracycline each at 10 μg/ml. Cellic™ CTec2 and HTec2 enzymes (a gift of Novozymes, Davis, CA, United States)lot numbers VCPI0004 and VHN00002, respectively) were used at a protein mass ratio of 3:1. In this paper, this mixture of CTec2 and HTec2 is abbreviated C/HTec2. The protein concentrations of CTec2 and HTec2 were taken as 130 mg/ml and 101 mg/ml, respectively [38]. Accellerase™ 1000 was a gift of Genencor/DuPont, Palo Alto, CA, United States) (lot number 1600844643; 69 mg protein/ml). Multifect™ Pectinase was a generous gift of Dupont/Danisco (Rochester, NY, United States). Digestion assays were performed in 96 deep-well plates at 50°C in a rotary incubator at 10 rpm for 72 hours with sampling every 24 hours [19]. Each reaction volume was 0.5 ml. Released Glc was measured colorimetrically with a glucose oxidase-peroxidase (GOPOD) reagent (Megazyme, Bray, Ireland). Assays were run in duplicate, sampled twice, and the Glc levels measured twice. Therefore, each data point represents the mean of eight values. Error bars represent +/−one standard deviation (SD) of the mean.

### Preparation and assay of extractives

Pretreated but unwashed biomass (equivalent to 1 g glucan according to Table 2) was washed sequentially with 20 ml of water, 20 ml ethanol, and 20 ml acetone on a Buchner funnel through Whatman #1 filter paper (GE Life Sciences, Piscataway, NJ, United States). The resulting extracted material solutions were dried at 50°C overnight and redissolved in 10 ml water. Glc content in the extractives was assayed directly as described above. The extractives were then digested to completion with C/HTec2 and re-measured for free Glc. The difference before and after enzymatic digestion was taken as Glc due to soluble oligosaccharides, with a small contribution from the C/HTec2 itself, which was subtracted.

**Table 2:** Effect of accessory enzymes on release of Glc from AHP-pretreated LQ by the 8-component synthetic enzyme mixture

	**Glc yield, % of maximum**
8-component alone	29.66 ± 0.74
+ Cip1	23.35 ± 3.15
+ Cip2	29.68 ± 0.03
+ Abf1	29.86 ± 0.51
+ Abf2	29.95 ± 0.28
+ AbfB	29.83 ± 0.69
+ XG	35.33 ± 0.34
+ GH12	27.48 ± 0.38
+ β-galactosidase	30.02 ± 1.76

Values are the means ± 1 SD, n =3. The 8-component mixture was used at 15 mg/gm glucan and each accessory enzyme was added at 5 mg/gm glucan.

Abf1, arabinosidase 1; Abf2, arabinosidase 2; AbfB, arabinosidase B; AHP, alkaline hydrogen peroxide; Cip1, cellulose-inducible protein 1; Cip2, cellulose-inducible protein 2; GH12, Cel12A; Glc, total glucose; LQ, lamb’s quarter; XG, xyloglucanase.

See Materials and Methods for the GenBank or JGI accession numbers.

Enzyme inhibition by the extractives was tested in a standardized assay containing CS at a concentration of 2 mg glucan/ml and a C/HTec2 loading of 1 mg/g glucan, as described below. Extractives derived from the equivalent of 1 mg glucan from unwashed biomass were tested per mg of glucan from corn stover.

### Optimization of synthetic 8-component enzyme mixtures

The *T. reesei* proteins used in the assays were produced by expression in *P. pastoris* as described, except for CBH1 (Cel7A), which was purchased from Megazyme (Bray, Ireland) [19,20]. The enzymes were concentrated and desalted but not otherwise purified. The DOE Joint Genome Institute (JGI) identifiers for the *T. reesei* proteins, and their alternate names and abbreviations, are CBH1 (cellobiohydrolase 1, Cel7A) [Tr_123989]; EG1 (endoglucanase, Cel7B) [Tr_122081]; CBH2 (Cel6A) [Tr_72567]; BG (β-glucosidase, Cel3) [Tr_76672]; EX2 (endo-β1,4-xylanase 2, Cel11) [Tr_123818]; EX3 (endo-β1,4-xylanase 3, Cel10) [Tr_120229]; BX (β-xylosidase, Cel3A) [Tr_121127]; GH61 (Cel61A) [Tr_73643]. Design-Expert™ software (Stat-Ease, Inc., Minneapolis, MN, United States) and the GLBRC Enzyme Platform (GENPLAT) were used for experimental design, analysis, and optimization of mixtures [19,20]. Enzyme hydrolysis was performed as described above. Per 500 μl assay, final glucan loading was 1 mg and enzyme loading was 15 μg.

### Accessory enzymes

Additional accessory enzymes were tested with the 8-component enzyme mixture at a final glucan loading of 1 mg and total enzyme loading of 20 mg/g glucan in 500 μl reactions. The accessory enzymes and their JGI identifier numberswere Abf1 (α-arabinosidase 1) [Tr_123279]; Abf2 (α-arabinosidase 2) [Tr_76210]; AbfB (α-arabinosidase b) [Tr_123283]; XG (xyloglucanase, Cel74A) [Tr_49081]; β-galactosidase, Cel35 [Tr_80240]; Cip1 [Tr_73638]; Cip2 [Tr_123940]; Cel12A (endoglucanase) [Tr_123232]. AxlA (α-xylosidase, Cel31) GenBank accession number [BK008484] from *A. niger* was produced by expression in *P. pastoris*[22,23].

## Abbreviations

AFEX: ammonia fiber expansion; AHP: alkaline hydrogen peroxide; C/HTec2: a 3:1 protein mass mixture of Cellic™ CTec2 and HTec2; CS: corn stover; Glc: glucose; Xyl: xylose; GR: goldenrod; LQ: lamb’s quarters; MW: milkweed; QA: Queen Anne’s lace.

## Competing interests

The authors declare that they have no competing interests.

## Authors’ contributions

DJ, ERA, AM, AW, JDW, and MSB made substantial contributions to the conception and design of the work, acquisition of data, and contributed to writing the manuscript. All authors read and approved the final manuscript.
